# Humoral immune response after different SARS-CoV-2 vaccination regimens

**DOI:** 10.1186/s12916-021-02231-x

**Published:** 2022-01-21

**Authors:** Ruben Rose, Franziska Neumann, Olaf Grobe, Thomas Lorentz, Helmut Fickenscher, Andi Krumbholz

**Affiliations:** 1 Institut für Infektionsmedizin, Christian-Albrechts-Universität zu Kiel und Universitätsklinikum Schleswig Holstein, Campus Kiel, Brunswiker Straße 4, D-24105 Kiel, Germany; 2 Labor Dr. Krause und Kollegen MVZ GmbH, Steenbeker Weg 23, D-24106 Kiel, Germany

**Keywords:** COVID-19, Vaccination schemes, Immunoglobulin G, Maturity process, Virus variants of concern, Virus neutralisation

## Abstract

**Background:**

The humoral immune response after primary immunisation with a SARS-CoV-2 vector vaccine (AstraZeneca AZD1222, ChAdOx1 nCoV-19, Vaxzevria) followed by an mRNA vaccine boost (Pfizer/BioNTech, BNT162b2; Moderna, m-1273) was examined and compared with the antibody response after homologous vaccination schemes (AZD1222/AZD1222 or BNT162b2/BNT162b2).

**Methods:**

Sera from 59 vaccinees were tested for anti-SARS-CoV-2 immunoglobulin G (IgG) and virus-neutralising antibodies (VNA) with three IgG assays based on (parts of) the SARS-CoV-2 spike (S)-protein as antigen, an IgG immunoblot (additionally contains the SARS-CoV-2 nucleoprotein (NP) as an antigen), a surrogate neutralisation test (sVNT), and a Vero-cell-based virus-neutralisation test (cVNT) with the B.1.1.7 variant of concern (VOC; alpha) as antigen. Investigation was done before and after heterologous (*n* = 30 and 42) or homologous booster vaccination (AZD1222/AZD1222, *n* = 8/9; BNT162b2/BNT162b2, *n* = 8/8). After the second immunisation, a subgroup of 26 age- and gender-matched sera (AZD1222/mRNA, *n* = 9; AZD1222/AZD1222, *n* = 9; BNT162b2/BNT162b2, *n* = 8) was also tested for VNA against VOC B.1.617.2 (delta) in the cVNT. The strength of IgG binding to separate SARS-CoV-2 antigens was measured by avidity.

**Results:**

After the first vaccination, the prevalence of IgG directed against the (trimeric) SARS-CoV-2 S-protein and its receptor binding domain (RBD) varied from 55–95% (AZD1222) to 100% (BNT162b2), depending on the vaccine regimen and the SARS-CoV-2 antigen used. The booster vaccination resulted in 100% seroconversion and the occurrence of highly avid IgG, which is directed against the S-protein subunit 1 and the RBD, as well as VNA against VOC B.1.1.7, while anti-NP IgGs were not detected. The results of the three anti-SARS-CoV-2 IgG tests showed an excellent correlation to the VNA titres against this VOC. The agreement of cVNT and sVNT results was good. However, the sVNT seems to overestimate non- and weak B.1.1.7-neutralising titres. The anti-SARS-CoV-2 IgG concentrations and the B.1.1.7-neutralising titres were significantly higher after heterologous vaccination compared to the homologous AZD1222 scheme. If VOC B.1.617.2 was used as antigen, significantly lower VNA titres were measured in the cVNT, and three (33.3%) vector vaccine recipients had a VNA titre < 1:10.

**Conclusions:**

Heterologous SARS-CoV-2 vaccination leads to a strong antibody response with anti-SARS-CoV-2 IgG concentrations and VNA titres at a level comparable to that of a homologous BNT162b2 vaccination scheme. Irrespective of the chosen immunisation regime, highly avid IgG antibodies can be detected just 2 weeks after the second vaccine dose indicating the development of a robust humoral immunity. The reduction in the VNA titre against VOC B.1.617.2 observed in the subgroup of 26 individuals is remarkable and confirms the immune escape of the delta variant.

**Supplementary Information:**

The online version contains supplementary material available at 10.1186/s12916-021-02231-x.

## Background

Since spring 2020, the pandemic caused by the *severe acute respiratory syndrome coronavirus 2* (SARS-CoV-2) [1] is ongoing and represents a global challenge. The availability of safe and effective vaccinations is seen as one of the most important pillars in containing the pandemic [2, 3]. Within a few months, intensive research activities led to the development of several highly effective SARS-CoV-2 vaccines [3–5]. In addition to the induction of cellular immunity, their administration should stimulate the formation of virus-neutralising antibodies (VNA) that bind to epitopes of the viral spike (S)-protein and its receptor binding domain (RBD) and, thus, prevent cell entry [3, 6, 7].

Four SARS-CoV-2 vaccines have received conditional approval in the European Union. These vaccines are based on two different technologies [8]. For the messenger ribonucleic acid (mRNA) vaccines from Pfizer/BioNTech (BNT162b2) and Moderna (mRNA-1273), the genetic information for the S-protein was optimised and the mRNA was packaged in liposomes. After inoculation, the muscle cells directly expressed this stable and highly immunogenic viral surface protein [2, 6]. In vector vaccines, replication-deficient human (Ad26.COV2; Janssen) or chimpanzee adenoviruses (ChAdOx1 nCoV-19/AZD1222, Vaxzevria; AstraZeneca, hereinafter referred to as AZD1222) are used to introduce the genetic information of the SARS-CoV-2 S-protein into the cells, followed by transcription of deoxyribonucleic acid into mRNA and expression of the S-protein [2, 6].

Due to the widespread use of these vaccines, rare and sometimes unexpected side effects have been reported. Particularly noteworthy are cases of immune thrombotic thrombocytopenia, which predominantly occurred in women under 50 years of age within 1 month after the initial vaccination with AZD1222 [5]. Many of these patients developed cerebral sinus venous thrombosis or splanchnic vein thrombosis and presented antibodies to platelet factor 4 but without previous exposure to heparin [5]. Due to this rare but serious side effect, AZD1222 is no longer unreservedly recommended by the Standing Vaccination Commission (STIKO) of the Robert Koch Institute for individuals under 60 years of age. The STIKO suggests that a vaccination with AZD1222 that has already started should be completed with an mRNA vaccine [9, 10]. Due to the sharp increase in the delta variant of concern (VOC; Pango-lineage [11] B.1.617.2) in Germany, the STIKO has revised its recommendations once more. Since July 1, 2021, all AZD1222 first vaccinated persons have been recommended to complete the second vaccination with an mRNA vaccine [12]. Animal experiments indicated very good humoral and cellular immunity after heterologous vaccination [13, 14]. There is, however, so far limited knowledge on the benefit of the heterologous vaccination scheme in humans. First results indicated a higher prevalence of short-lived side effects following the heterologous boost dose compared to the homologous counterpart [15]. Meanwhile, a few studies have been published for the immunogenicity of the AZD1222/mRNA vaccine regimen [16–22].

In this report, we compare the SARS-CoV-2-specific immunoglobulin G (IgG) response after heterologous immunisation with that elicited by homologous vaccination schedules. We also focus on the developing anti-SARS-CoV-2 IgG avidity as a parameter for IgG maturity and binding strength. Finally, we investigate the development of VNA against two prevalent VOCs. The various methods described are being used to thoroughly study vaccine-induced humoral immune response magnitude and surrogate efficacy. We believe that the results obtained in this study will help to better understand the effects and possibly benefits of a heterologous vaccination regimen.

## Methods

The anti-S and anti-RBD IgG response after heterologous immunisation with a SARS-CoV-2 vector vaccine as prime and an mRNA vaccine as boost was compared to that after homologous vaccination with vector or mRNA vaccines. This setting also includes monitoring of IgG avidity and of virus-neutralising capacities. Forty-seven female and twelve male vaccinees with a median age of 31 years (age span 18–61 years) were recruited for this study and gave their informed consent. The prior SARS-CoV-2 infection status was not queried. Since it was not clear in advance how many individuals could be recruited for the study, we did not estimate the required number of cases. Rather, all vaccinees who declared their willingness to participate were included. Forty-two of them received a heterologous immunisation scheme (*N* = 40, AZD1222/BNT162b2; *N* = 2, AZD1222/mRNA-1273), while nine and eight vaccinees received a homologous scheme of the vector vaccine AZD1222 or the mRNA vaccine BNT162b2, respectively. The first blood sample was taken immediately before the second vaccination and the second about 2 weeks later (median 14–16 days, time span 10–34 days, Table 1). Several individuals only contributed samples before or after vaccine boost and do not have matched data. The ethics committee of the medical faculty of the Christian-Albrechts-Universität zu Kiel (Kiel, Germany) approved the study design (D467/20, 16.04.2020, amendment 02.02.2021). We examined the early humoral immune response (other samples obtained a few days to weeks after the initial immunisation with AZD1222) of most of the subjects in a previous study. In addition, sera obtained from three vaccinees after the initial immunisation with BNT162b2 (*N* = 2) and AZD1222 (*N* = 1) have already been tested in frame of the previous study [23]. In this respect, we consider it justified to include these individuals (and few sera) in the present report and to demonstrate the results before and after the second vaccination.

**Table 1:** Individuals included in this study

Study groups	Number of individuals after 1st/2nd vaccination	Median age in years	Age or age span in years	Gender (female/male)	Time (median) from 1st vaccination up to 1st serum sampling in days	Time (median) from 2nd vaccination up to 2nd serum sampling in days
*Heterologous vaccination scheme*						
AZD1222/BNT162b2	28/40	27	18–56	32/8	69	15
AZD1222/BNT162b2 (subgroup)†	-/9	43	23–56	7/2	Not applicable	14
AZD1222/mRNA-1273	2/2	‡	24, 45	2/0	64, 69 (‡)	10, 14 (‡)
*Homologous vaccination scheme*						
AZD1222	8/9	41	23–61	6/3	69	16
BNT162b2	8/8	35	23–51	7/1	34	14

†After the 2nd vaccination, several sera were also tested for the presence of virus-neutralising antibodies against the SARS-CoV-2 delta variant of concern (B.1.617.2). For this purpose, individuals from the group of heterologous vaccinations whose age and gender largely corresponded to those with homologous vaccinations were chosen. This subgroup is separately presented. ‡Calculating the median does not make sense if there are two values

### Anti-SARS-CoV-2-specific IgG immunoassays

The sera were tested with the SERION ELISA agile SARS-COV-2 IgG assay (S-protein as antigen; Institut Virion\Serion GmbH, Würzburg, Germany; negative: < 10 U/ml, borderline range 10–14 U/ml, positive ≥15 U/ml; linearity range 3–250 U/ml) and the Abbott SARS-CoV-2 IgG II Quant assay (RBD as antigen; Abbott, Wiesbaden, Germany; cut-off = 50 AU/ml) as described previously [23]. In addition, the LIAISON® SARS-CoV-2 Trimeric S IgG assay (cut-off = 33.8 Binding Antibody Units (BAU)/ml; linearity range 4.81 to 2080 BAU/ml) was included as a further assay on a LIAISON® XL system (both Diasorin S.p.A, Saluggia, Italy). According to the manufacturer, this quantitative chemiluminescence immunoassay detects IgG directed against the trimeric S-protein and has an excellent clinical sensitivity and specificity of 98.7% and 99.5%, respectively. The high diagnostic value of this test has also been demonstrated in a recent seroprevalence study [24]. The results of the three IgG assays were given in BAU per ml, using the manufacturer’s conversion factors, which were based on measurements of the WHO International Standard Anti-SARS-CoV-2 Immunoglobulin (NIBSC code 20-136) [25]. As in our previous studies, we rate the borderline test results of the SERION ELISA agile SARS-COV-2 IgG assay as positive [23, 26]. If a serum had an anti-SARS-CoV-2 IgG concentration above the linearity range, this sample was 1:10 (SERION ELISA agile SARS-COV-2 IgG assay) or 1:20 (LIAISON® SARS-CoV-2 Trimeric S IgG assay) diluted in the manufacturer’s dilution buffer and then measured again. The concentration was then recalculated. Since the linearity of the assay is no longer given after dilution, the measured values determined in this way may not entirely correspond to reality. We consider this effect to be negligible.

### Anti-SARS-CoV-2 IgG immunoblots including measurement of IgG avidities

The sera were tested in the *recom*Line SARS-CoV-2 IgG assay using the Dynablot Plus system together with a BLOTrix reader and the recomScan software (all from Mikrogen GmbH, Neuried, Germany) as reported previously [23, 26]. This immunoblot consists of a nitrocellulose strip on which the recombinant SARS-CoV-2 nucleoprotein (NP) as well as the S1-and RBD-subunits of its S-protein are separately spotted. In addition, the blot carries the recombinant NPs of four seasonal human coronaviruses (HCoVs 229E, NL63, OC43, and HKU1). By comparing the IgG binding to the SARS-CoV-2 antigens in the presence and absence of the avidity reagent, the binding strength was automatically determined and assessed [23, 26]. The results were used to assign IgG avidity to four categories: no avidity detectable (= 0), low avidity (= 1), intermediate avidity (= 2) and high (= 3) avidity [23].

### Measurement of SARS-CoV-2-neutralising antibodies

The sera were examined for their virus-neutralising capacities. First, a surrogate assay was used by strictly following the manufacturer’s instructions (TECO® SARS-CoV-2 Neutralisation Antibody ELISA; TECOmedical AG, Sissach, Switzerland). In this competitive assay, the human angiotensin-converting enzyme 2 (ACE-2) was attached to the solid phase while peroxidase-conjugated RBD was present in the liquid phase. If the human serum contained RBD-specific antibodies, binding of RBD to ACE-2 was prevented. Hence, after washing steps, the colour reaction turned out to be weaker compared to a RBD-antibody-free serum sample. According to the manufacturer, it is assumed from an inhibition of RBD to ACE-2 binding ≥20% that VNA are present [23].

Second, dilutions of each serum were tested in triplicate in an in-house 96-well format Vero-cell-based neutralisation assay (cVNT) as previously reported [23]. In brief, 2.5 × 10^4^ Vero cells (order no. 605372, CLS Cell Lines Service GmbH, Eppelheim, Germany) were seeded per well and incubated at 37 °C under standard conditions. On the next day, sera were heat-inactivated (56 °C for 30 min) and diluted in a cell culture medium (1:10, 1:20, 1:40, 1:80, 1:160, 1:320, 1:640, and 1:1280). The latter consists of Dulbecco’s modified Eagle’s medium supplemented with 3.7 g/l NaHCO3, 4.5 g/l glucose, 2 mM l-glutamine, and 1% (v/v) of Pen-Strep-Fungi mix containing 10,000 U/ml penicillin, 10 mg/ml streptomycin, and 25 μg/ml amphotericin B (all reagents from Bio&SELL GmbH, Feucht, Germany). Then, 25 μl of the serum dilution was mixed with 25 μl of virus suspension containing 50 plaque-forming units (pfu) of either an own VOC B.1.1.7 strain (alpha, from January 2021) or an own VOC B.1.617.2 strain (delta, from June 2021; both viruses were obtained after cultivation of material obtained from the upper respiratory tract of SARS-CoV-2 patients in Vero cells as described previously [27]). The resulting 50 μl was incubated for 1 h at 37 °C. Meanwhile, Vero cells were washed with phosphate-buffered saline (PBS, Bio&SELL). Then, 50 μl of the virus-serum dilutions was pipetted on the prepared cells followed by 1 h of incubation on a shaker at room temperature. Thereafter, 50 μl of a fresh cell culture medium supplemented with 20% foetal calf serum (v/v) was added per well and plates were incubated for 4 days under standard conditions. Then, cells were fixed by addition of 4% (w/v) paraformaldehyde in PBS and stained with an aqueous solution of 0.1% (w/v) crystal violet and 20% (v/v) methanol. The cytopathic effect (CPE) formation was compared with an untreated cell control (medium only) and a viral control (50 pfu). A serum dilution > 1:10 (titre) that prevented CPE formation in at least two of three wells compared with the viral control was valued as containing neutralising antibodies. Recent data indicate that titres from around 1:100 are associated with a high vaccine effectiveness of > 80% [28]. When an exact titre could not be provided by the eye, the geometric mean of the two adjacent titres was calculated [23, 26].

### Data evaluation and statistical calculations

Data were statistically analysed by the help of the GraphPad Prism version 9.1.2 software (GraphPad Software, San Diego, CA, USA). In most cases, the Kruskal-Wallis test, an adjusted, non-paired and non-parametric test, was applied. The Wilcoxon test, a paired non-parametric test, was chosen to compare the median VNA titre differences against VOC B.1.1.7 and VOC B.1.617.2. To compare the frequencies of the measured anti-SARS-CoV-2 IgG avidity indices as a function of the vaccination scheme used, Fisher’s exact test was applied, adjusted by the Bonferroni correction for multiple testing. The level of significance was generally set at *P* = 0.05. Furthermore, we calculated the Spearman correlation coefficient to demonstrate the correlation between separate data sets. We used a simple logistic regression to determine the probability of detecting VNA with our cVNT as a function of the anti-SARS-CoV-2 IgG or sVNT results.

## Results

This study included 59 individuals. Nine and eight of them received a homologous immunisation with AZD1222 or BNT162b2, respectively, while 42 received a heterologous vaccination. The composition of the study groups including median age, age range, gender, and median time of blood collection in days is shown in Table 1.

**Fig. 1:**
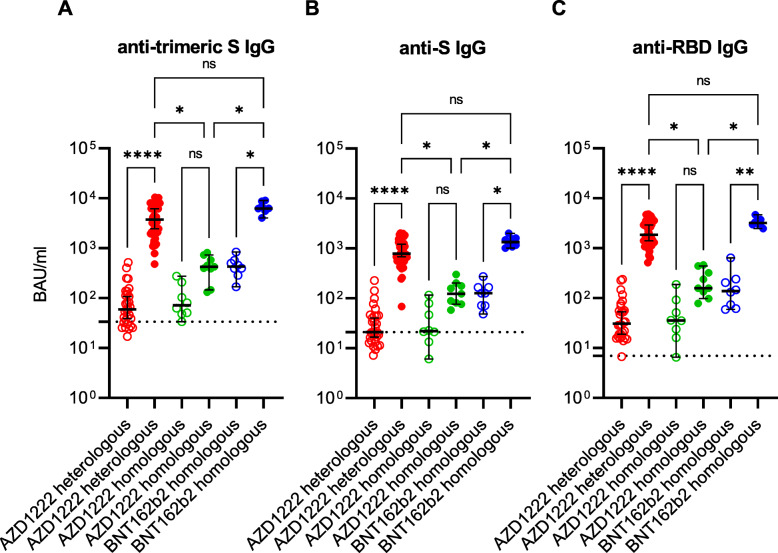
Anti-SARS-CoV-2 immunoglobulin G (IgG) response in Binding Antibody Units (BAU) per millilitre (ml) after first (empty circles) and second (filled circles) immunisation with the vector vaccine AZD1222 or the messenger ribonucleic acid (mRNA)-based vaccines BNT162b2 or mRNA-1273. The cut-offs for positivity (i.e. presence of anti-SARS-CoV-2 IgG including borderline results) of anti-trimeric spike (S) IgG assay **(A)**, of the anti-S IgG assay **(B)** and of the anti-receptor binding domain (RBD) IgG assay **(C)**, respectively, are marked by dashed lines. The median and the 95% confidence interval were calculated for each group. Ns non-significant; *p < 0.05; **p < 0.01; ****p < 0.0001 (Kruskal-Wallis test)

After the first immunisation, all individuals who received an mRNA vaccine developed anti-(trimeric)-S and anti-RBD-IgG. The vaccinees who received the AZD1222 had a response rate of 55.3% (anti-S IgG), 76.3% (anti-trimeric S IgG), and 94.7% (anti-RBD IgG), respectively. After administration of the second dose, the IgG response rate reached 100% in all groups. The median of anti-SARS-CoV-2 IgG concentrations varied between 20.9 BAU/ml (anti-S IgG; first vaccination in the AZD1222 heterologous group) and 6240 BAU/ml (anti-trimeric S IgG; second vaccination in the BNT162b2 homologous group). After the second vaccine dose, an increase in median anti-SARS-CoV-2 IgG concentrations was observed in all three study groups and in all assays. Compared to the median anti-SARS-CoV-2 IgG concentrations after the vector vaccine AZD1222 was administered twice, the corresponding concentrations were 6 to 12 times higher after a heterologous vaccination and even 11 to 20 times higher when compared to the homologous BNT162b2 vaccination scheme (Fig. 1; Additional file 1: Table S1).

**Fig. 2:**
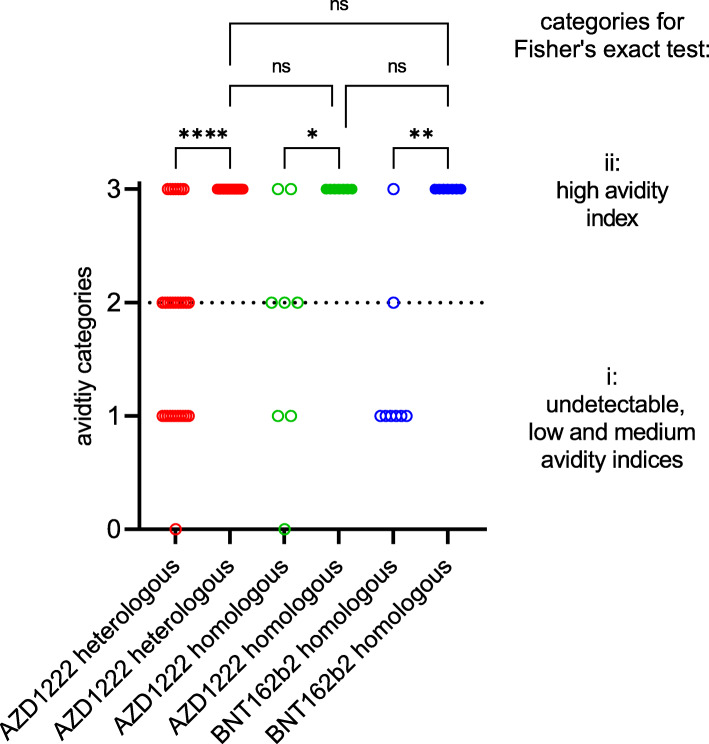
Development of anti-SARS-CoV-2 immunoglobulin G avidities after first (empty circles) and second (filled circles) immunisation with the vector vaccine AZD1222 or the messenger ribonucleic acid (mRNA)-based vaccines BNT162b2 or mRNA-1273. The measured IgG avidities were assigned to the four categories of undetectable (0), low (1), intermediate (2) and high (3) index. The significance of the distribution differences was calculated between the two groups of undetectable, low and intermediate (i) on the one hand and high (ii) avidity indices on the other. Ns not significant; *p < 0.02; **p < 0.003; ****p < 0.00003 (Bonferroni-adjusted Fisher’s exact test)

Next, we examined the presence of IgG directed against the separate NPs of seasonal HCoVs and SARS-CoV-2 as well as against the S1 and RBD of SARS-CoV-2 in an immunoblot. Between 19.6 and 38.0% of the vaccinees had IgG antibodies that were directed against the NPs of seasonal HCoVs, while none showed IgG reactivity against the NP of SARS-CoV-2. The second vaccine dose resulted in a 100% prevalence of anti-S1 and anti-RBD IgG antibodies (Additional file 1: Fig. S1). Furthermore, development of anti-SARS-CoV-2 IgG avidity was recorded. After the first vaccination, the majority of the measured IgG avidity indices were in the low to intermediate range. In contrast, high IgG avidities were consistently observed after administration of the second vaccine dose (Fig. 2; Additional file 1: Table S1).

**Fig. 3:**
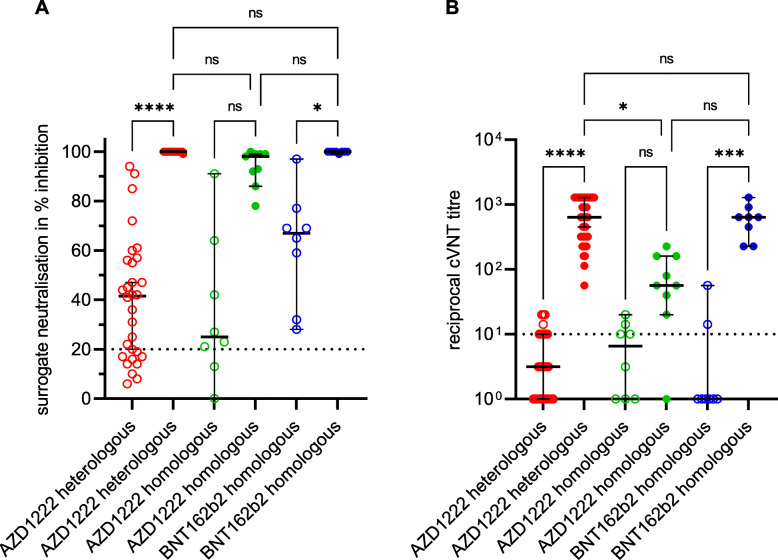
Development of SARS-CoV-2 neutralising antibodies (VNA) after first (empty circles) and second (filled circles) immunisation with the vector vaccine AZD1222 or the messenger ribonucleic acid (mRNA)-based vaccines BNT162b2 or mRNA-1273. A surrogate neutralisation assay (**A**) and a Vero-cell-based virus-neutralisation test (cVNT) using the SARS-CoV-2 variant of concern B.1.1.7 (alpha) strain (**B**) were applied to measure the VNAs. The assay cut-offs are indicated by dashed lines. The median and the 95% confidence interval were calculated for each group in **A** and **B**. Ns non-significant; *p < 0.05; ***p < 0.001; ****p < 0.0001 (Kruskal-Wallis test)

**Fig. 4:**
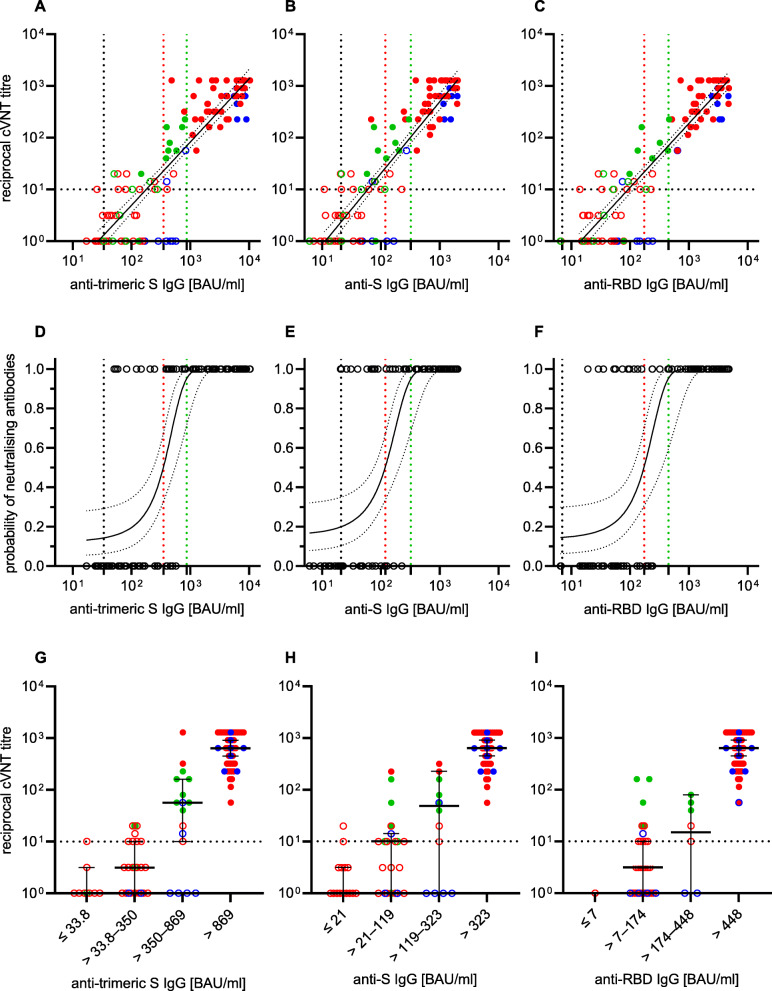
Anti-SARS-CoV-2 immunoglobulin G (IgG) response in Binding Antibody Units (BAU) per millilitre (ml) after first (open circles) and second (filled circles) immunisation with the vector vaccine AZD1222 (green), the messenger ribonucleic acid (mRNA)-based vaccine BNT162b2 (blue) and after a heterologous vaccination scheme, starting with AZD1222, followed by an mRNA-based vaccine boost (BNT162b2 or mRNA-1273; red) with regard to the detection of virus-neutralising antibodies (VNA). The latter were measured in a Vero-cell-based neutralisation test (cVNT) using the SARS-CoV-2 variant of concern B.1.1.7 (alpha). Cut-off values for positivity of the anti-trimeric spike (S) IgG assay (**A**), anti-S IgG assay (**B**) and anti-receptor binding domain (RBD) IgG assay (**C**), respectively, and the cVNT cut-off value for the presence of VNA are indicated by black dashed lines. The Spearman correlation coefficients of log(reciprocal titre) were calculated with 0.86, 0.86 and 0.88, respectively. The probability of detecting VNA at a given BAU/ml in the anti-SARS-CoV-2 IgG assays was calculated by logistic regression (**D**–**F**): VNA were present in 95% of samples when IgG concentrations of 886 BAU/ml (anti-trimeric S IgG), 323 BAU/ml (anti-S IgG) and 448 BAU/ml (anti-RBD IgG), respectively, were measured (green dashed lines; 95% confidence intervals (CI) 59.4 to 99.6%). Vertical black dashed lines represent the threshold values set by the manufacturers of the antibody assay; red dashed lines represent the BAU/ml concentrations (anti-trimeric S IgG: 350 BAU/ml; anti-S IgG: 119 BAU/ml; anti-RBD IgG: 174 BAU/ml) with a 50% probability of VNA detection. The distribution of the cVNT titres, the medians, and the 95% CIs between the three plotted thresholds (dashed black, red and green lines in **A**–**F**) are shown (**G**–**I**)

**Fig. 5:**
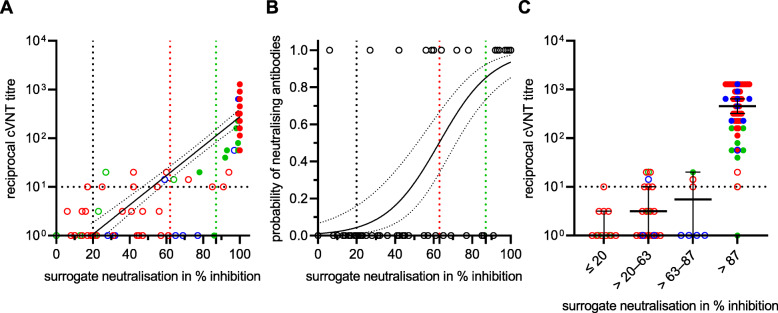
Correlation of the surrogate neutralisation test (sVNT) results with results obtained by the laboratory-developed Vero-cell-based virus-neutralisation test (cVNT) using a B.1.1.7 strain as antigen (**A**). The Spearman correlation coefficient of log(reciprocal titre) was calculated with 0.88; empty circles*:* first vaccination; filled circles: second vaccination; red: heterologous vaccination with AZD1222/mRNA; green: homologous vaccination with AZD1222; blue: homologous vaccination with BNT162b2. Probability of detecting virus-neutralising antibodies (VNA) with the cVNT at a given percentage inhibition of sVNT calculated by logistic regression **(B)**; e.g. at 20% inhibition (black dashed line), 63% inhibition (red dashed line), and at 87% inhibition of sVNT (green dashed line), the probabilities of detecting VNA with cVNT are 4% (95% confidence interval (CI) 1–16%), 50 % (95% CI 34–66 %) and 85% (95% CI 73–92%), respectively. The distribution of the cVNT titres, their medians, and their 95% CIs between the three plotted thresholds (dashed black, red and green lines in **A**, **B**) are shown (**C**)

The virus-neutralising properties of the sera were examined with two different assays. A so-called surrogate neutralisation test was used to investigate the extent to which the anti-RBD antibodies that may be present in the serum are able to prevent the binding of this S-protein subunit to the human receptor ACE-2. In addition, a laboratory-developed virus-neutralisation test was used, which is based on a VOC B.1.1.7 strain as the antigen. While the sVNT classified the majority of the sera as virus-neutralising after the first vaccination, most of these samples in the cVNT were below the cut-off from which virus neutralisation can be assumed. With both methods, however, an increase in the level of VNA could be detected after a second vaccination. There were also marked differences between the three vaccination groups, both in the degree of inhibition (sVNT) and in the level of VNA titres (cVNT). Vaccinees who had received the vector vaccine only had 11-fold lower median VNA titres (1:57) compared to individuals immunised heterologously with AZD1222 and an mRNA vaccine (1:640) or homologously with BNT162b2 (1:640). In contrast, the median percentage inhibition of sVNT reached a similar level in all three groups (Fig. 3; Additional file 1: Table S1). The quantitative results of the anti-SARS-CoV-2 IgG assays showed an almost perfect correlation to the VNA titres using VOC B.1.1.7 as antigen in the cVNT (Spearman correlation coefficient of 0.86 to 0.88; Fig. 4A–C). In addition, from a certain anti-SARS-CoV-2 IgG concentration, it could be assumed with a probability of 95% that VNA are present. The anti-SARS-CoV-2 IgG BAU/ml concentrations required for this differed slightly between the three assays (323 BAU/ml in the anti-S IgG test; 448 BAU/ml in the anti-RBD IgG test; 886 BAU/ml in the anti-trimeric S IgG test). However, there is a large overlap of samples that are virus-neutralising or not at a similar IgG concentration (Fig. 4D–F). The correlation between the measured values of cVNT (VOC B.1.1.7 as antigen) and sVNT was comparable (Spearman correlation coefficient of 0.88) to those calculated for the three IgGs. It is noticeable that especially non-neutralising sera (VNA titre ≤1:10) were overestimated in the sVNT. This is evidenced by the fact that the cut-off set by the manufacturer was only associated with a 4% probability of detecting VNA in our cVNT (Fig. 5).

**Fig. 6:**
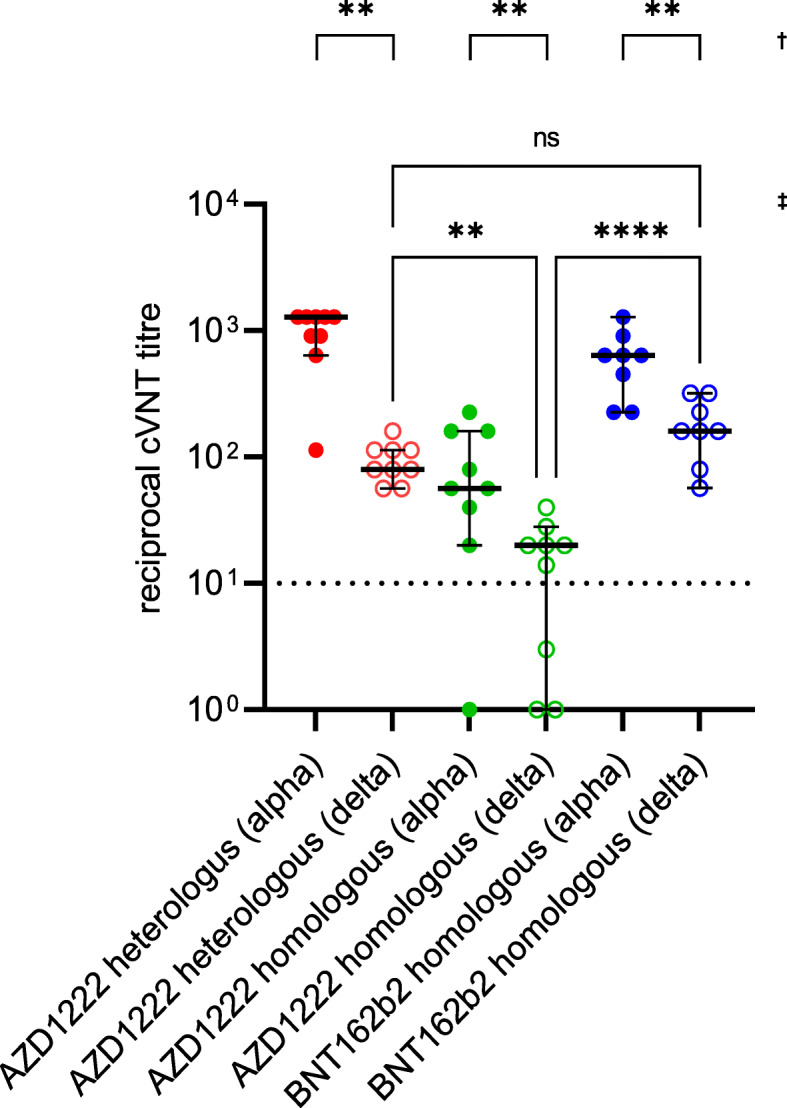
Presence of virus-neutralising antibodies (VNA) against the SARS-CoV-2 variants of concern B.1.1.7 (alpha, filled circles) and B.1.617.2 (delta, empty circles) after the second immunisation. Sera from 26 age- and gender-matched individuals who received a heterologous (AZD1222/BNT162b2, *n* = 9) or a homologous vaccination scheme (AZD1222/AZD1222, *n* = 9; BNT162b2/BNT162b2, *n* = 8) were tested (see Table 1). An individual VNA titre > 1:10 was defined as neutralising in our Vero-cell-based virus-neutralisation test (cVNT). †The significance of the median VNA titre differences against B.1.1.7 and B.1.617.2 was calculated using the Wilcoxon test (**p < 0.01). ‡Comparison of the median VNA titre differences achieved with different immunisation schemes against B.1.617.2 (Kruskal-Wallis test; ns not significant; **p < 0.01; ****p < 0.0001)

A subset of age- and gender-matched sera (Table 1) obtained after the second immunisation were also tested in the cVNT for the presence of VNA against VOC B.1.617.2 (delta). In comparison to B.1.1.7, all three vaccination groups exhibited significantly lower median VNA titres (AZD1222/BNT162b2: 1:1280 vs. 1:80, 16-fold lower; AZD1222/AZD1222: 1:57 vs. 1:20, 2.9-fold lower; BNT162b2/BNT162b2: 1:640 vs. 1:160, 4-fold lower) when B.1.617.2 is used as the antigen. If the vaccination schedule is used as a comparator, those vaccinated homologously with the vector vaccine had significantly lower median VNA titres (1:20) than those homologously vaccinated with BNT162b2 (1:160). This difference was also found for the heterologous vaccination regime (1:80). In contrast, the median VNA titres between those vaccinated homologously with BNT162b and those vaccinated heterologously (AZD1222/BNT162b2) exhibited no significant difference (Fig. 6; Additional file 1: Table S1).

## Discussion

Due to the rare but serious side effects after administration of the vector vaccine (AZD1222), in spring 2021, the STIKO recommended that people under the age of 60 should complete vaccinations that had already been started with a vector vaccine with an mRNA vaccine [9, 10]. This recommendation was extended on July 1, 2021, to all who had already received a primary vaccination with AZD1222 [12]. In the first quarter of this year, however, only a few animal experimental data sets were available on the immunological outcome of the proposed heterologous vaccination scheme [13, 14]. Several studies have now appeared on the immunogenicity of such immunisation schemes in humans [16–22, 29].

In this report, we compared the development of the humoral immune response after homologous and heterologous vaccination with different methods. To the best of our knowledge, we are one of the first research groups to investigate the anti-delta VOC neutralising effect of sera after completing the heterologous immunisation regime.

After the first vaccination, the majority of individuals developed anti-trimeric-S, anti-S, and anti-RBD IgG antibodies, respectively. However, their concentrations varied between the three study groups. The results are in line with our previous study [23]. It is evident that the anti-SARS-CoV-2 IgG concentrations are not comparable between the three assays either. This is probably due to the different antigen preparations. In order to make valid statements about the kinetics of the antibody concentration in individuals or to compare the antibody response between different groups, identical assays should preferably be used; alternatively, the inclusion of reference standards could be useful.

The second immunisation resulted in higher concentrations in all three groups. It was noticeable that significantly higher anti-SARS-CoV-2 IgG concentrations were detected after a second vaccination with an mRNA vaccine than after the vector vaccine AZD1222 was administered again. The increase in anti-S and anti-RBD IgG concentrations after a second vaccination with an mRNA vaccine confirms our previous study [23]. Due to the recommended vaccination interval of 10 to 12 weeks, we did not yet have any data on the development of the SARS-CoV-2-specific IgG antibodies after the second administration of a vector vaccine [23]. The lack of anti-SARS-CoV-2 NP-specific IgG antibodies in all vaccinees can be interpreted as an indication that they had no COVID-19 infection and were therefore to be regarded as immunologically naive before immunisation [23]. It is known that vaccinations lead to particularly high anti-SARS-CoV-2 IgG concentrations in convalescents [30]. For these individuals, the recommendation is that they should receive a vaccine dose about 6 months after they have been infected [10]. Less than half of the vaccinees had IgG antibodies to the NPs of seasonal coronaviruses, which is lower than the prevalence reported by other authors for adults [31].

After the first vaccination, nearly all individuals exhibited only low to intermediate avid anti-SARS-CoV-2 IgG, while after the second vaccine dose IgG of high avidity appeared in all cases. These results confirm and expand the existing knowledge on the development of highly avid anti-SARS-CoV-2 IgG after a second vaccination with an mRNA vaccine [32]. In line with this, VNA titres > 1:10 against the previously prevalent SARS-CoV-2 VOC B.1.1.7 (alpha) were observed after second immunisation which confirms our recent study [23]. Marked differences in median VNA titres were observed between individuals re-vaccinated with an mRNA vaccine and those re-vaccinated with the vector vaccine. The anti-SARS-CoV-2 IgG concentrations obtained with three preparations of the viral S-protein correlated well with the presence and level of VNA titres using B.1.1.7 as the antigen in our cVNT. This observation is in line with a recently published study from Finland [33] and suggests that these standardised and easy-to-perform commercial tests are useful for measuring vaccine-induced responses. High anti-SARS-CoV-2 IgG concentrations were associated with the presence of high VNA titres against VOC B.1.1.7 (alpha). However, anti-SARS-CoV-2 IgG concentrations that indicate the presence of protective VNA cannot be defined across the board. These depend on the cVNT and the viral antigen used in it. Basically, the sVNT also showed the titre increase and evaluated all sera as virus-neutralising after the second vaccination with values close to 100% inhibition of RBD binding to ACE-2. However, it is again noticeable that sera that are not or only weakly SARS-CoV-2 neutralising in the in-house cVNT are categorised as neutralising in the sVNT, which supports our previous proposal to increase the cut-off of this surrogate assay [23]. Compared to the admittedly very conservative in-house cVNT, a cut-off of over 80% binding inhibition would be desirable. Due to the lack of standardisation of the widespread cVNTs for the detection of VNA against SARS-CoV-2, this recommendation only applies to our laboratory and cannot be generalised.

A particularly interesting point is the significantly reduced capacity for neutralising the SARS-CoV-2 delta variant (VOC B.1.617.2) in vitro using a cVNT. We observed this in the subgroup of 26 age- and gender-matched individuals regardless of the immunisation regime. In addition, three of nine vaccinees who had received two doses of AZD1222 presented low or undetectable VNA against this VOC, which is considered to be 60% more transmissible than alpha [34]. The SARS-CoV-2 delta VOC is known to have accumulated a number of mutations in the S-protein. These enable continued good binding to the cellular ACE-2 receptor, but at the same time lead to the viral S-protein being less efficiently recognised by antibodies [34]. The significantly lower VNA titres compared to the alpha VOC, which we and others [34, 35] observed, corroborate the suspicion of an immunescape of the delta VOC. The results of our cVNT for B.1.1.7 suggest that a single vaccination would not be sufficient to induce measurable VNA against B.1.617.2.

The data presented by us on the antibody response after heterologous SARS-CoV-2 vaccination are consistent with the few available clinical studies [16–22, 29]. In June 2021, a randomised study from Spain has already demonstrated that the heterologous vaccination scheme is suitable for generating a robust immune response. Unfortunately, this very extensive work did not include a control group of individuals who received two immunisations with the vector vaccine [21]. A recent preprint reports significant higher anti-S antibody concentrations in a group of 26 individuals who first received an AZD1222 vaccination followed by re-vaccination with BNT162b2 compared to 14 individuals that were vaccinated twice with BNT162b2. However, these results were obtained with a total antibody assay which does not discriminate between IgG and acute phase immunoglobulin M. The VNA titres against chimeric vesicular stomatitis viruses carrying the S-proteins of SARS-CoV-2 VOCs B.1.1.7 (alpha) or B.1.351 (beta) as antigens, respectively, were markedly higher in the AZD1222/BNT162b2 group compared to the BNT162b2/BNT162b2 recipients, while data after homologous vaccination with the vector vaccine were not presented [16]. A further study, however, which also includes results from individuals who were vaccinated twice with AZD1222, came to the same conclusion [22]. In another investigation [18], differences in the anti-S IgG concentrations and VNA titres were not observed after heterologous vaccination (AZD1222/mRNA) or after homologous vaccination with an mRNA vaccine while both parameters were significantly lower after homologous vaccination with the vector vaccine. These results are in agreement with our data even if only one IgG assay and one sVNT were used by this research group [18]. The data of a single-blind randomised British study, in which the four possible vaccine combinations of AZD1222 and BNT162b2 were compared with one another, are very interesting and promising. These researchers report about 9-fold higher geometric mean anti-S IgG concentrations in sera from heterologous AZD1222/BNT162b2 vaccinees compared to individuals immunised twice with AZD1222 [19]. This largely corresponds to our results and to data of a current preprint, which describes the immune response after administration of combinations of vector (Ad26.COV2 - S, Janssen) and mRNA (BNT162b2, mRNA-1273) vaccines [29]. In a study from Sweden, markedly higher anti-S and anti-RBD IgG concentrations were observed after heterologous vaccination (AZD1222/mRNA-1273) compared to the homologous AZD1222 scheme. Likewise, significantly higher VNA titres were measured both against a wild-type SARS-CoV-2 and against a VOC strain B.1.351 (beta). Two doses of AZD1222, however, did not induce potent VNA titres against the beta VOC [20] as also observed by others [22]. One investigation reports the development of high IgG avidity after completion of a homologous (AZD1222/AZD1222; BNT162b2/BNT162b2) or heterologous SARS-CoV-2 (AZD1222/BNT162b2) immunisation scheme. While there were no qualitative differences in the development of IgG avidity between both groups, the AZD1222/BNT162b2 vaccinees developed a significantly higher relative avidity index [17]. In our IgG avidity assay, we cannot measure such gradual differences. For the AZD1222/AZD1222 group, lower anti-SARS-CoV-2 IgG concentrations as well as lower B.1.1.7/B.1.351-pseudovirus-neutralising titres were reported [17].

It is not yet sufficiently clear why homologous vaccination with the AZD1222 vector vaccine leads to lower anti-SARS-CoV-2 IgG concentrations and VNA titres. A possible explanation could be the immune response to the adenovirus vector backbone (so-called antivector immunity [3]).

The work presented by us contributes to a better understanding of the humoral immunogenicity of the heterologous SARS-CoV-2 vaccination regimen. With various assays, we monitored the development of anti-S-specific IgG antibodies and make statements about their binding strength as an expression of maturity. In addition, with a commercial and an in-house test, we showed that VNA can be detected after the second vaccination and that VNA titres vary in dependence of the viral antigen.

Important limitations of our report are (i) the heterogeneity of the study groups; (ii) the marked difference in time between the first immunisation and sampling which may impair data comparability; (iii) the small group size of individuals who received a homologous vaccination scheme; (iv) the subjects, who are predominantly in younger to middle adulthood; (v) the lack of information on the durability of the detected antibodies; and (vi) the missing consideration of cellular and innate immunity after immunisation. Therefore, no statements can be made about the need for further booster vaccinations. In addition, our data on anti-SARS-CoV-2 IgG concentrations do not allow any valid predictions about the degree of protection against natural SARS-CoV-2 infection.

## Conclusions

The administration of a vector vaccine followed by an mRNA vaccine boost resulted in a strong humoral immune response, comparable to that after two immunisations with an mRNA vaccine. Regardless of the vaccination scheme, all individuals developed highly avid anti-SARS-CoV-2 IgGs as well as VNA against a B.1.1.7 strain (alpha VOC) after the second immunisation. However, the generally lower neutralising titres against the B.1.617.2 strain, which were observed in the subgroup of 26 vaccinees demonstrate a partial immune escape of the delta VOC. While these results require further confirmation, they suggest that adapting the vaccine to current virus variants may be useful.

## Supplementary Information

**Additional file 1.** Table S1: Vaccine-induced humoral immunity. Fig. S1: Nucleoprotein (NP) specific immune response.

## Data Availability

The manuscript and Additional file 1 contain all relevant data from the study.

## Abbreviations

ACE-2: Angiotensin-converting enzyme 2
AZD1222: Vector vaccine from AstraZeneca (ChAdOx1, Vaxzevria)
BAU: Binding Antibody Units
BNT162b2: Messenger ribonucleic acid vaccine from Pfizer/BioNTech
cVNT: Vero-cell-based neutralisation test
IgG: Immunoglobulin G
m-1273: Messenger ribonucleic acid vaccine from Moderna
mRNA: Messenger ribonucleic acid
NP: Nucleoprotein
RBD: Receptor binding domain
SARS-CoV-2: *Severe acute respiratory syndrome coronavirus 2*
S-protein: Spike protein
STIKO: Standing Vaccination Commission of the Robert Koch Institute
sVNT: Surrogate neutralisation test
VNA: Virus-neutralising antibodies
VOC: Variant of concern
WHO: World Health Organization
