# Supplementary material for: Humoral immune response after different SARS-CoV-2 vaccination regimens
Source: BMC Med. 2022 Jan 21;20:31. doi: 10.1186/s12916-021-02231-x (PMC8776512; doi:10.1186/s12916-021-02231-x)
Supplement: Supplementary file 1 — Additional file 1. Table S1: Vaccine-induced humoral immunity. Fig. S1: Nucleoprotein (NP) specific immune response. [file 12916_2021_2231_MOESM1_ESM.pdf]

Table S1: Vaccine-induced humoral immunity. Anti-SARS-CoV-2 immunoglobulin G (IgG) response after first and second immunisation with the vector vaccine AZD1222 or the messenger ribonucleic acid (mRNA)-based vaccines BNT162b2 and mRNA-1273. The IgG concentration is given in Binding Antibody Units (BAU) per ml. The median and the 95% confidence intervals (CI) were calculated for each group. The table also contains the anti-SARS-CoV-2 IgG avidity results. Furthermore, the virus neutralising titres determined in the surrogate neutralisation test (sVNT) or in the Vero-cell based virus neutralisation test (cVNT) using a B.1.1.7 (alpha) or B.1.617.2 (delta) strain as antigens are given.

	**AZD1222 heterologous 1st vaccination**	**AZD1222 heterologous 2nd vaccination**	**AZD1222 homologous 1st vaccination**	**AZD1222 homologous 2nd vaccination**	**BNT162b2 homologous 1st vaccination**	**BNT162b2 homologous 2nd vaccination**
**Number of tested sera**	30	42	8	9	8	8
**anti-trimeric S IgG**						
Minimum (BAUml)	17	480	34	131	168	4030
25% Percentile (BAU/ml)	32	1973	49	267	312	5693
Median (BAU/ml)	59	3750	72	424	428	6240
75% Percentile (BAU/ml)	125	7280	182	656	551	8200
Maximum (BAU/ml)	517	10400	274	811	835	9010
95%CI median, lower limit (BAU/ml)	39	2460	34	146	168	4030
95%CI median, upper limit (BAU/ml)	107	6170	274	729	835	9010
**anti-S IgG**						
Minimum (BAUml)	7	68	6	58	48	1011
25% Percentile (BAU/ml)	14	619	15	79	71	1093
Median (BAU/ml)	21	783	22	123	126	1334
75% Percentile (BAU/ml)	45	1328	71	190	166	1589
Maximum (BAU/ml)	226	2024	116	299	272	2002
95%CI median, lower limit (BAU/ml)	17	676	6	76	48	1011
95%CI median, upper limit (BAU/ml)	40	1213	116	203	272	2002
**anti-RBD IgG**						
Minimum (BAUml)	7	513	7	78	59	2470
25% Percentile (BAU/ml)	17	1249	18	116	65	2993
Median (BAU/ml)	31	1859	36	157	139	3195
75% Percentile (BAU/ml)	60	3141	82	380	232	3715
Maximum (BAU/ml)	239	4795	187	462	638	4676
95%CI median, lower limit (BAU/ml)	19	1404	7	98	59	2470
95%CI median, upper limit (BAU/ml)	53	2927	187	439	638	4676
**IgG-Avidity†**						
Number of tested sera	30	36	8	7	8	7
IgG avidity index 0 - 2	24	0	6	0	7	0
IgG avidity index 3	6	36	2	7	1	7
**sVNT**						
Number of tested sera	30	42	8	9	8	8
Minimum (% inhibition)	6	99	0	78	28	99

	AZD1222 heterologous 1st vaccination	AZD1222 heterologous 2nd vaccination	AZD1222 homologous 1st vaccination	AZD1222 homologous 2nd vaccination	BNT162b2 homologous 1st vaccination	BNT162b2 homologous 2nd vaccination
25% Percentile (% inhibition)	17	100	15	89	39	100
Median (% inhibition)	42	100	25	98	67	100
75% Percentile (% inhibition)	56	100	59	99	75	100
Maximum (% inhibition)	94	100	91	100	97	100
95%CI median, lower limit (% inhibition)	20	100	0	86	28	99
95%CI median, upper limit (% inhibition)	47	100	91	99	97	100
**cVNT (alpha)**						
Minimum (titre)	<1:10	1:57	<1:10	<1:10	<1:10	1:226
25% Percentile (titre)	<1:10	1:320	<1:10	1:30	<1:10	1:283
Median (titre)	1:3[†]	1:640	1:7[†]	1:57	<1:10	1:640
75% Percentile (titre)	1:10	1:1280	1:13	1:160	1:11	1:839
Maximum (titre)	1:20	1:1280	1:20	1:226	1:57	1:1280
95% CI of median, lower limit (titre)	<1:10	1:453	<1:10	1:20	<1:10	1:226
95% CI of median, upper limit (titre)	1:10	1:1280	1:20	1:160	1:57	1:1280
**cVNT**	**AZD1222 heterologous 2nd vaccination**		**AZD1222 homologous 2nd vaccination**		**BNT162b2 homologous 2nd vaccination**	
**Variant of SARS-CoV-2**	**alpha**	**delta**	**alpha**	**delta**	**alpha**	**delta**
Number of tested sera	9	9	9	9	8	8
Minimum (titre)	1:113	1:57	<1:10	<1:10	1:226	1:57
25% Percentile (titre)	1:773	1:68	1:30	1:2	1:283	1:100
Median (titre)	1:1280	1:80	1:57	1:20	1:640	1:160
75% Percentile (titre)	1:1280	1:113	1:160	1:24	1:839	1:297
Maximum (titre)	1:1280	1:160	1:226	1:40	1:1280	1:320
95% CI of median, lower limit (titre)	1:640	1:57	1:20	<1:10	1:226	1:57
95% CI of median upper limit (titre)	1:1280	1:113	1:160	1:28	1:1280	1:320

† The avidity of the anti-SARS-CoV-2 IgG was not tested in all sera. For example, if an individual already presented highly avid IgG antibodies after the first vaccination, the avidity was not determined again after the second vaccination.

Titres <1:10 and ≤1:10 were included in the statistics as log(1) and log(3.2) respectively for mathematical reasons.

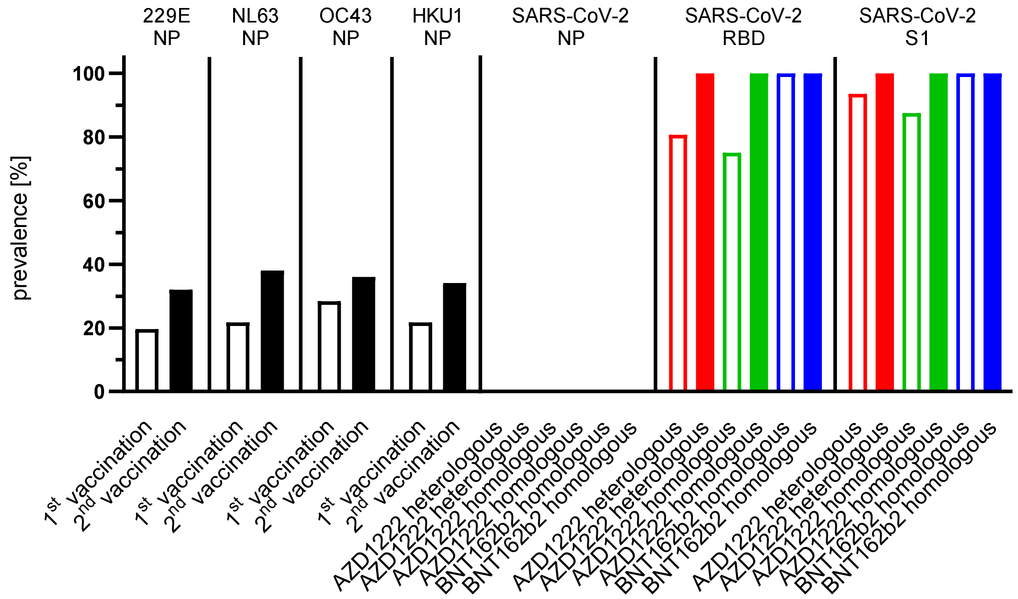

Fig. S1: Nucleoprotein (NP) specific immune response. Pattern of immunoglobuline G (IgG) reactivity against the separate NPs of seasonal human coronaviruses (HCoVs 229E, NL63, OC43, and HKU1) and the NP of SARS-CoV-2 as well as the Spike-protein subunit S1 and the receptor binding domain (RBD). For HCoVs, the prevalence of the different vaccination regimens is summarised after the first and second vaccination and given as a percentage. Reactivity was recorded after first (empty bars) and second immunisation (filed bars) with the vector vaccine AZD1222 or the messenger ribonucleic acid (mRNA)-based vaccines BNT162b2 or mRNA-1273.
